# On the Prevention of Uterine Hemorrhage Arising after Delivery, from Atony of the Uterus

**Published:** 1848-08

**Authors:** J. Christie

**Affiliations:** Aberdeen


*On the prevention of Uterine Hæmorrhage, arising after delivery, from atony of the Uterus.* By J. Christie, M. D. Aberdeen.--Flooding is, at all times, a serious and alarming accident. Whether it occurs in the earlier months of pregnancy, in the latter, or subsequent to delivery, it demands the instant adoption of decisive means, in order to suppress it, or even to restrain it within the bounds consistent with the safety, and the future health of the patient.

In numerous instances it is obviously impossible to foresee its occurrence subsequent to delivery, consequently, preventive measures of a special kind, and having reference, by anticipation, to such an accident, is, in these cases, out of the question. But on the other hand, cases do occur where the anticipation of hæmorrhage is well founded, and then, the accoucheur ought to arm himself with, and put in force, all the resources of science, and all the appliances of art, in an effort to prevent it, from the instant that its probable occurrence assumes a tangible shape in his mind.

A labour may progress well, and its termination may promise a happy and unclouded issue to the thankful and delighted mother,—a promise also shared in by the accoucheur; yet experience tells us, that all these anticipations are often swept away in an instant, by the sudden and unlooked-for occurrence of flooding.

To foresee all such cases is utterly impossible. No experience, however great, or knowledge and observation, however profound on the one hand, or accurate on the other, can enable one to anticipate with certainty the sudden and dangerous loss of blood subsequent to delivery. There are, however, instances in which it is to be dreaded, even from the commencement of labour, and as the remarks I mean to present to the profession on this subject, will be confined to such cases, I quote the following one, taken from my notes of them, almost at random.

Mrs. S——, aged 38, a woman of a lax, leuco-phlegmatic habit of body, was taken in labour, early in the morning of the 24th December, 1847.

At ten o'clock I was summoned to her bedside; and, on making a vaginal examination, I found the os uteri enlarged to the size of a crown-piece, soft, and very dilatable. The pains were natural and apparently sufficiently powerful, as, in the course of half an hour, the os uteri was dilated to at least twice the size already noted, and the bag of membranes had begun to press on the floor of the pelvis. But although everything was thus seemingly progressing towards a happy result, the woman herself expressed great anxiety as to the ultimate event of her labour, the dread of flooding almost completely unnerving her, although she confessed the labour was not in itself unbearable, or the subject of apprehension.

On enquiring into the cause of this dread, I learned that this was her seventh labour, and that the three immediately preceding deliveries had been followed by dangerous flooding, which, in each instance, had taken place after her medical attendant had left the house. Nothing of an unusual nature had marked the three first confinements. In her last confinement, the hæmorrhage had been very profuse; and it was only after the uterus had been emptied of large masses of coagulated blood, and stimulated to contract by the presence of the hand in its cavity, that the flooding was suppressed.

The habit of my patient, both in a physical and moral sense, clearly indicated under the circumstances described, the necessity of adopting means to prevent the recurrence of an event so much and justly dreaded; and which, there was every reason to believe, would as readily recur as on the three former occasions.

The first indication to be fulfilled was, obviously, to reassure my patient's mind,—to quiet the apprehension so conducive to the very accident which was the subject of fear; and, having, in some measure, succeeded in this, the second was to employ, *previous* to delivery, such means as were calculated to prevent hæmorrhage, by securing the firm and permanent contraction of the uterus in the accouchée.

The woman being of a lax habit of body, and the abdominal parietes in consequence relaxed, and moreover still further weakened by the repeated distension of pregnancy, the support of an abdominal bandage, even before the termination of labour, was an evident desideratum, and, therefore, it was at once applied.

The next object was to act, if possible, directly on the uterus, for although labour was progressing in the most favourable manner, so far merely as its mechanism was concerned, the almost certain occurrence of post partum hæmorrhage was to be provided against; and, to accomplish this, nothing seemed more likely than the exhibition of ergot of rye. As yet, however, it was not deemed advisable to administer it, there being nothing in the progress of the labour itself requiring its exhibition. The sole object was to secure the contraction of the uterus after the termination of the labour; and, accordingly, it was thought proper to delay the administration of the ergot, until the process of parturition should be so far advanced, as to promise a speedy issue in delivery. In the course of three quarters of an hour the distended membranes came to dilate the vulva; and, having ruptured during a pain, the head was to be felt in contact with the perineum. The next pain pushed the head so far down as to distend the perineum to a considerable extent; and, now, the labour being so near a termination, an infusion of the ergot, containing the bi-borate of soda in solution, was exhibited on the subsidence of the pain. In ten minutes the child was born; and in a quarter of an hour subsequently the placenta was removed from the vagina, where it had lain since the expulsion of the fœtus, the same pain that expelled the fœtus having thrown it off and expelled it from the uterus.

On placing the hand on the abdomen over the region of the uterus, it was found to be firmly contracted into a ball, as it were, about the size of the child's head. In two or three minutes it became softer and larger, but immediately afterwards it again contracted, and although this alternate state of relaxation and contraction continued for at least twenty minutes, the time during which the hand was applied to the abdomen, there was no greater discharge of blood than is usual. The hand was now removed and the abdominal bandage fastened firmly, a common towel folded two or three times on itself, being laid under it, and over the fundus of the uterus. Previous to delivery the bandage was two or three times adjusted and fastened, so as to support, without impeding, the action of the abdominal muscles.

The labour having so far terminated favourably, I remained in the house for an hour and a half, but on the expiry of that period, and there being no flooding, I took my leave, after having once more satisfied myself that the uterus maintained its contracted state, by examining the hypogastric region. From this time the case progressed most favourably, and the woman made a rapid and satisfactory recovery.

*Remarks.*—I could have easily cited a number of cases in proof of the efficacy of the treatment adopted in this instance, with a view to prevent hæmorrhage depending on atony of the uterus subsequent to delivery. But the case narrated involves everything of importance, in a practical point of view; and, to multiply illustrations of similar and equally fortunate results, could serve no other purpose than to repeat the same outline of leading facts, a course, to follow which, would be as useless as it would be tedious. It may be said, that even had hæmorrhage supervened, a fatal, or even a dangerons result, might not have followed, as, in most instances, flooding is amenable to the ordinary and established modes of treatment. But the object of my treatment was *prevention*; it was eutirely conservative in its nature; and, although, in general, flooding is controlled by active and decisive measures, I had no wish that my patient should run the gauntlet of a chance, where appearances were so much against her. Besides, had the means employed been of no service, they did not preclude the recourse, in the event of flooding, to other suitable aids in the suppression of hæmorrhage.

In another point of view the exhibition of ergot of rye in such a case appears to me to be most beneficial, namely, in not only preventing hæmorrhage, but, also, in preventing the severe after-pains, which almost universally annoy women who have borne a large family, and who, at the same time, are of a lax habit of body.

The *modus operandi* of the drug must, of course, be obvious to all; and, as it acts in preventing flooding, by stimulating the uterus to contract, it follows from thence, that after-pains are also prevented by the firm and permanent contraction thus induced.

The use of the bandage as a subsidiary means must also be taken into account; still, I am satisfied, from a tolerably wide experience, that the ergot is most to be relied upon. In fact, it is but seldom I apply a bandage, previous to delivery, although I am well aware it is the common practice of many experienced accoucheurs; but in cases similar to the one I have given, I have only in one or two other instances thought it necessary to use it.

I have often seen after-pains of the most severe and tormenting nature,—nay, I have even seen them merge in metritis—where the ergot was used in order to rouse the uterus to action, but this was in cases where rigidity or some other obstructive cause rendered labour tedious, and where the contractile power of the uterus was, as it were, worn out by the continued and *stormy* action it excited. Indeed, so much am I averse to the use of ergot of rye in exciting the uterus to action, that in most instances, especially in prima-paræ, I prefer the use of the forceps. Cautiously and tenderly used nothing can be safer, particularly if the mechanism of labour be *thoroughly* understood. But to digress on this point, would be to enter on a discussion more suited to an elaborate review of the subject, than to an incidental notice in a slight sketch of this kind.

Before concluding, however, I would express my conviction, that no greater boon could be conferred on obstetric medicine, than settling the value of ergot of rye, by a discovery of the cases where it can be appropriately and safely exhibited, both as regards the mother and child. Much as has been written on the subject, it requires an enlightened and patient revisal; and, I trust, the value and importance of ergot in obstetric cases, suited to its exhibition, will find a competent and favourably situated observer in the ranks of the profession in Britain.—*British Record of Obstetric Medicine, &c.*

